# Induction of protective and therapeutic anti-pancreatic cancer immunity using a reconstructed MUC1 DNA vaccine

**DOI:** 10.1186/1471-2407-9-191

**Published:** 2009-06-18

**Authors:** Yefei Rong, Dayong Jin, Wenchuan Wu, Wenhui Lou, Danshong Wang, Tiantao Kuang, Xiaoling Ni, Xinyu Qin

**Affiliations:** 1 Department of General surgery, Zhongshan Hospital, Fudan University, Shanghai, PR China

## Abstract

**Background:**

Pancreatic cancer is a common, highly lethal disease with a rising incidence. MUC1 is a tumor-associated antigen that is over-expressed in pancreatic adenocarcinoma. Active immunotherapy that targets MUC1 could have great treatment value. Here we investigated the preventive and therapeutic effect of a MUC1 DNA vaccine on the pancreatic cancer.

**Methods:**

MUC1-various tandem repeat units(VNTR) DNA vaccine was produced by cloning one repeat of VNTR and inserting the cloned gene into the pcDNA3.1. In the preventive group, female C57BL/6 mice were immunized with the vaccine, pcDNA3.1 or PBS; and challenged with panc02-MUC1 or panc02 cell. In the therapeutic group the mice were challenged with panc02-MUC1 or panc02 cell, and then immunized with the vaccine, pcDNA3.1 or PBS. The tumor size and the survival time of the animals were compared between these groups.

**Results:**

The DNA vaccine pcDNA3.1-VNTR could raise cytotoxic T lymphocyte (CTL) activity specific for MUC1. In the preventive experiment, the mice survival time was significantly longer in the vaccine group than in the control groups (*P *< 0.05). In the therapeutic experiment, the DNA vaccine prolonged the survival time of the panc02-MUC1-bearing mice (*P *< 0.05). In both the preventive and therapeutic experiments, the tumor size was significantly less in the vaccine group than in the control groups (*P *< 0.05). This pcDNA3.1-VNTR vaccine, however, could not prevent the mice attacked by panc02 cells and had no therapeutic effect on the mice attacked by panc02 cells.

**Conclusion:**

The MUC1 DNA vaccine pcDNA3.1-VNTR could induce a significant MUC1-specific CTL response; and had both prophylactic and therapeutic effect on panc02-MUC1 tumors. This vaccine might be used as a new adjuvant strategy against pancreatic cancer.

## Background

Pancreatic adenocarcinoma is the fourth leading cause of cancer death in the world. However recent advances in diagnostics, staging, and therapy in pancreatic adenocarcinoma have not resulted in significant improvements in the long-term survival [1]. Median survival for all patients does not exceed 2 years with a 5-year survival rate less than 20% [2]. Therefore new approaches are necessary to improve the outcome of patients suffering from pancreatic cancer.

Immunotherapy has recently become a feasible tumor specific therapy. MUC1 is a tumor-associated antigen that is over-expressed in pancreatic adenocarcinomas [3]. MUC1 is a transmembrane molecule whose major extracellular domain is composed of various tandem repeat units (VNTR) consisting of 20 amino acids (GVTSAPDTRPAPGSTAPPAH) and is the most specific epitope for tumor immunotherapy [4,5]. The MUC1-specific antibodies have been detected in the sera of breast, pancreatic, and colon carcinoma patients, indicating that MUC1 could induce humoral immune responses [6,7]. Von Mensdorff-Pouilly et al. reported that breast carcinoma patients who had natural humoral responses against MUC1 had a higher probability of disease-free survival [8]. In addition, MUC1-specific cytotoxic T lymphocytes (CTLs) have also been found in breast, pancreatic, and ovarian carcinoma patients [9,10].

Induction of MUC1-specific immune responses has already been reported in mice and humans. Mice immunized with the MUC1-VNTR peptides [11], MUC1-mannnan fusion protein [12], or dendritic cells transfected with MUC1 cDNA [13] could develop both humoral and cellular immune responses and suppress the growth of MUC1-expressing tumors. MUC1 vaccines have also been used in several clinical trials wherein cancer patients were immunized with either synthetic peptides, or DCs transfected with MUC1 cDNA. Although MUC1-specific antibodies and/or CTLs were detected in some patients, they were not adequate to generate effective anti-tumor immunity [14,15]. Furthermore, most of the vaccines were used for prevention of the tumor; and few of them were used for tumor therapy. Consequently, it is necessary to develop new vaccination protocols to induce strong anti-tumor immune responses that are applicable for the therapy of the pancreatic cancer.

In this paper, we showed that a MUC1 DNA vaccination strategy succeeded in suppressing pancreatic adenocarcinoma in C57BL/6 mice.

## Methods

### Plasmid DNA vaccine construction and manufacturing

The pcDNA3.1-VNTR plasmid encoding a human MUC1 cDNA was constructed as described by Wu [16]. This MUC1 cDNA encoded 20 amino acids (GVTSAPDTRPAPGSTAPPAH), which was one repeat of VNTR. An optimized Kozak sequence and an hMCP-1 sequence were engineered to the start codon region. Transient transfection demonstrated that pcDNA3.1-VNTR could induce expression of human MUC1 in Cos-7 cells.

The pcDNA3-MUC1 plasmid encoding 22 VNTR was kindly provided by Dr. Finn [17].

### Mice and tumor cells

The C57BL/6 mice were bred in the Animal Lab center (Medical school of Jiaotong University, Shanghai). The facility was approved by the Association for Assessment and Accreditation of Laboratory Animal Care International, and all procedures were carried out in accordance with the Guidelines and Regulations for Use and Care of Animals in Fudan University. The panc02 is a highly tumorigenic murine pancreatic tumor cell line with ductal morphology that was derived in 1984 from a methylcholanthrene-induced tumor growing in a C57BL/6 female mouse(kindly provided by MD Anderson Cancer Center)[18]. It produces rapidly growing local tumors after s.c. inoculation. The MUC1 cDNA (pcDNA3-MUC1) was transfected into the panc02 cell line using Lipofectamine 2000 Reagent (Invitrogen, Mannheim, Germany) according to the manufacturer's specifications. After selection with G418, clones that expressed MUC1 (panc02-MUC1-C and panc02-MUC1-F) were obtained by the limiting dilution method. Control clone panc02-Neo (abbreviated as panc02) was obtained by panc02 cells transfected with the pcDNA3 vector alone. These cell lines were maintained in RPMI1640 medium containing 10% heat-inactivated bovine serum (FBS).

### Vaccination protocol and tumor challenge studies

#### Tumor protection

The mice were divided randomly into 3 groups (n = 15). A total of 100 μg pcDNA3.1-VNTR in 100 μl of PBS was injected into the anterior tibialis muscle of the mice (every 2 weeks, 3 times). The mice treated with either the empty plasmid pcDNA3.1 or PBS were used as the control. Seven days after the third immunization the mice were inoculated with panc02-MUC1 at the interderm of the left anterior leg armpit (1 × 10^6^/each mouse). The tumor development in individual mice was monitored every 2–3 days and the tumor size (in mm^3^) was calculated by the following formula: 0.5 × length (mm) × width (mm)^2^. The survival time (until death or when the tumor volume was over 1,000 mm^3^) after the tumor challenge was recorded.

#### Tumor therapy

Mice in therapeutic experiments were divided into 4 groups (n = 9), and received s.c. injection of 1 × 10^6 ^panc02-MUC1 or panc02 cells in 200 μl of PBS 4 days before the immunization of pcDNA3.1-VNTR, pcDNA3.1 or PBS. The intramuscular DNA administrations were repeated on the ninth and thirteenth days after the first vaccination. The tumor sizes of the mice were recorded every 2–3 days to draw a tumor growth curve. The survival time (until death or when the tumor volume was over 1,000 mm^3^) after the tumor challenge was recorded to evaluate the therapeutic effect of the DNA vaccine.

#### Cytotoxicity assay

Cytotoxicity of CTL against the target cells was detected by a non-radioactive lactate dehydrogenase (LDH)-releasing assay (CytoTox96, Promega, USA). Mice were vaccinated with pcDNA3.1-VNTR, pcDNA3.1 plasmids (100 μg each plasmid) or PBS three times at biweekly intervals. Splenocytes were harvested 7 days after the third immunization. Then the splenocytes were isolated and samples were pooled and resuspended in RPMI complete media (RPMI 1640, 100 U/ml penicillin, 100 μg/ml streptomycin, 10%FBS). The effector cells were added to the target cells (panc02-MUC1 or panc02) in a 96-well plate at effector: target (E: T) ratios of 80:1, 40:1, 10:1. The procedure was carried out according to the manufacturer's instructions. Cytotoxicity was calculated using the following formula:

Where E: the experimental LDH release in effector plus target cell co-cultures; Se: the spontaneous release by effector cells alone; St: the spontaneous release by target cells alone; Mt: the maximal release by target cells.

#### Anti -MUC1 antibody

Serum samples were obtained from mice via retro-orbital bleeding 5 days after the third vaccination. Samples were analyzed for the presence of anti-MUC1 antibodies by ELISA. Microtiter plates were coated with 50 ng/well MUC1 protein (VNTR) at 4°C overnight. The plates were blocked (0.5% porcine gelatin, 4% BSA in PBS) and serum samples were assayed at 1:200 dilution. Anti-MUC1 antibodies were detected with horseradish peroxidase (HRP)-labeled anti-mouse IgG and developed with tetramethylbenzidine-stable. Absorbance was read at 450 nm. 1/titer values were scored as positive for the presence of MUC1 antibodies if the OD readings were at least three-times over PBS control wells.

### Statistical analysis

Data are presented as mean ± S.E.M. Statistical differences between two groups were evaluated by the unpaired Student's *t*-test. One-way ANOVA was used for the comparisons among three groups. The survival time was calculated by the Kaplan-Meier method. The survival rates were compared by the log-rank test (SPSS v11.0). *P*-values less than 0.05 were considered significant.

## Results

### Monoclonal cell line verifications

#### Western blot analysis

**Figure 1:**
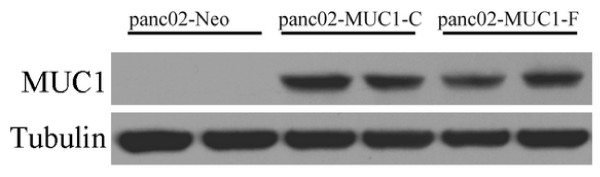
**Monoclonal cell line western blot analysis**. Panc02-MUC1-C and panc02-MUC1-F were transfected with pcDNA3-MUC1; while panc02-Neo was transfected with pcDNA3. The cell line panc02-MUC1-C and panc02-MUC1-F expressed the MUC1 protein.

The monoclonal cell line panc02-MUC1-C and panc02-MUC1-F could express the MUC1 protein while the control cell line panc02-Neo showed no expression of the MUC1 (Figure 1). The cell line panc02-MUC1-C was named pan02-MUC1 and used in subsequent experiments, while the cell line transfected with pcDNA3 was named panc02-Neo (abbreviated as panc02).

#### Immunostaining

**Figure 2:**
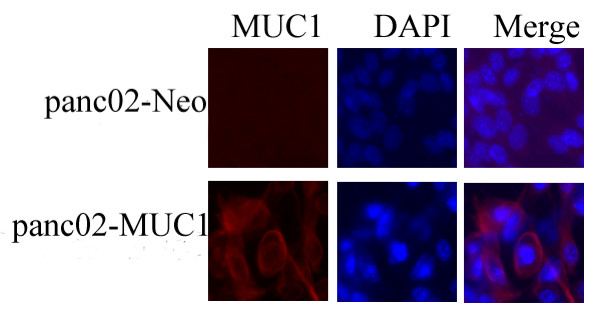
**Monoclonal cell line Immunostaining analysis**. Panc02-MUC1 could express MUC1 proteins especially on the membrane; panc02-Neo had no expression of MUC1.

MUC1 was expressed in the monoclonal cell line panc02-MUC1, especially on the surface of the cells, however the panc02 cell line showed no expression of MUC1 (Figure 2).

### Protective immune response

**Figure 3:**
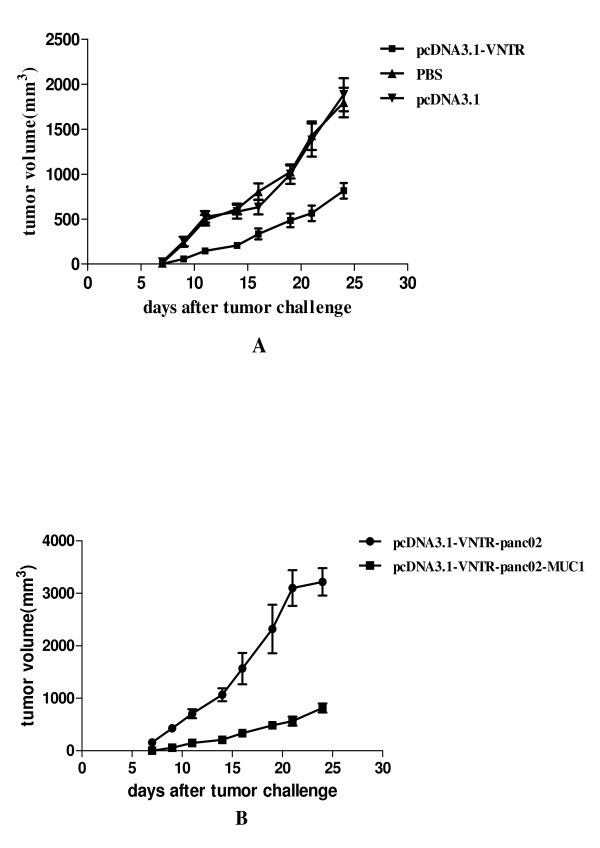
**Tumor protection in mice immunized with pcDNA3.1-VNTR**. **A:**Mice were immunized with either pcDNA3.1-VNTR, or the empty vector pcDNA3.1, or PBS, as indicated in the legends, followed by challenge with panc02-MUC1 tumor cells (n = 15 mice/group). Each plasmid was administrated at 100 μg doses. The mean tumor volume of the mice immunized with pcDNA3.1-VNTR was significantly smaller than that in the mice in the other two groups (PBS and pcDNA3.1) (mean ± S.E.M. *P *< 0.0001). **B: **Mice were immunized with pcDNA3.1-VNTR, and then challenge with panc02 tumor cells (n = 6). The mean tumor volume in these mice was significantly different from that in mice immunized with pcDNA3.1-VNTR and challenged with panc02-MUC1 (mean ± S.E.M. *P *< 0.0001).

To study the protective immune response of pcDNA3.1-VNTR vaccine against pancreatic cancer, the mice were inoculated with pcDNA3.1-VNTR three times before the mice were challenged with 1 × 10^6^panc02-MUC1 or panc02. As shown in Figure 3A, vaccination with pcDNA3.1-VNTR resulted in a significant protection compared to the empty vector and PBS (*P *< 0.0001 by one-way ANOVA). Furthermore, the protection against the tumor was MUC1-specific, since the mice that were immunized with pcDNA3.1-VNTR, followed by challenging with panc02 tumor cells were not protected (Figure 3B, *P *< 0.0001 by *t*-test). These results demonstrated that the pcDNA3.1-VNTR vaccine could induce MUC1-specific tumor protection.

**Figure 4:**
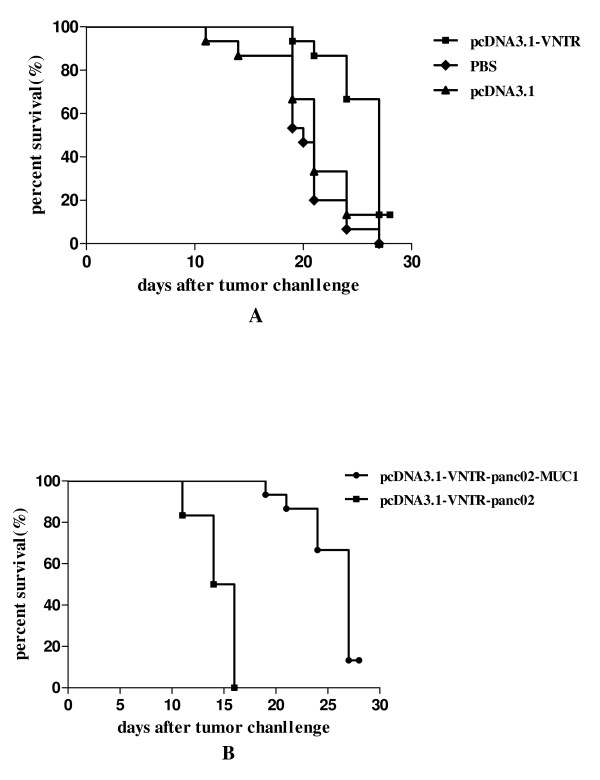
**Effect of DNA vaccine on survival of tumor challenged mice**. **A**: Mice were immunized with pcDNA3.1-VNTR, the empty vector pcDNA3.1, or PBS, as indicated in the legends, followed by challenge with panc02-MUC1 tumor cells. Mice were monitored for tumor development until a tumor volume of over 1000 mm^3 ^or death. As shown in the figure, the mice immunized with pcDNA3.1-VNTR survived longer compared to the mice in the other two groups (*P *< 0.05 by Log-rank test). **B: **Mice immunized with pcDNA3.1-VNTR, followed by challenge with panc02 tumor cells had a much shorter survival time compared with the mice challenged with panc02-MUC1(*P *< 0.05 by Log-rank test).

Long-term survival of pcDNA3.1-VNTR-vaccinated mice was assessed. Immunized mice were tumor-challenged and monitored for tumor growth until death or until the tumor volume was over 1000 mm^3^. As show in Figure 4A, the mice immunized with pcDNA3.1-VNTR were protected better against the panc02-MUC1 tumor cells than the mice in the pcDNA3.1 and PBS groups, because the mice in this group survived longer in comparison with the mice in the other two groups. Moreover this effective protection was MUC1-specific, because the survival time of the mice immunized with pcDNA3.1-VNTR and challenged with panc02 tumor cells did not increase (Figure 4B).

### Anti -MUC1 antibody

**Figure 5:**
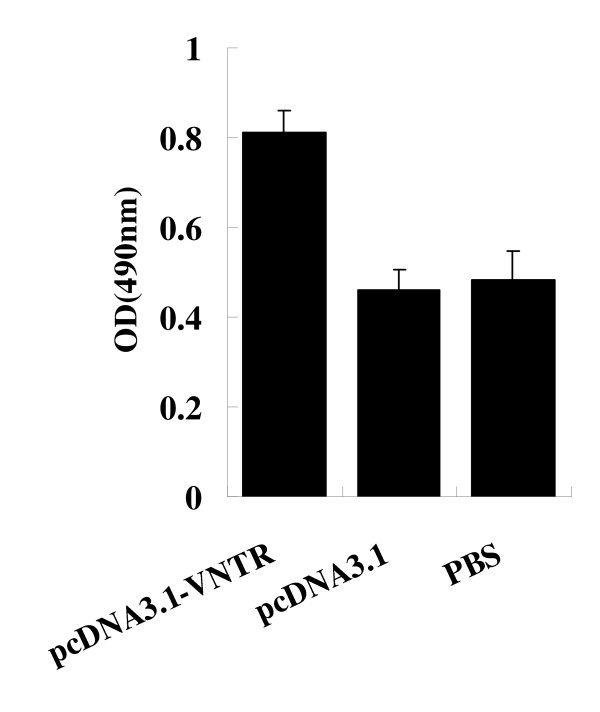
**Induction of specific antibody responses**. Mice were immunized with pcDNA3.1-VNTR, pcDNA3.1 or PBS and sera were harvested 5 days after the third immunization. The MUC1-specific IgG antibodies in sera of different groups were determined at a 1:200 dilution by ELISA. As shown in figure, relatively higher titres of anti-MUC1 antibodies were detected in the serum of pcDNA3.1-VNTR-vaccinated mice compared to that in the sera of the empty vector and PBS vaccinated groups (*P *< 0.05 by one-way ANOVA). Gtr6gthjyu.

Because specific antibodies also play a role in antitumor immunity, we measured the anti-MUC1 titres in the serum of immunized mice by ELISA, 5 days after the third time immunization. As shown in Figure 5, relatively higher titres of anti-MUC1 antibodies were detected in the serum of pcDNA3.1-VNTR-vaccinated mice than the empty vector and PBS vaccinated groups (*P *< 0.05 by one-way ANOVA)

### Induction of Cytotoxicity responses

**Figure 6:**
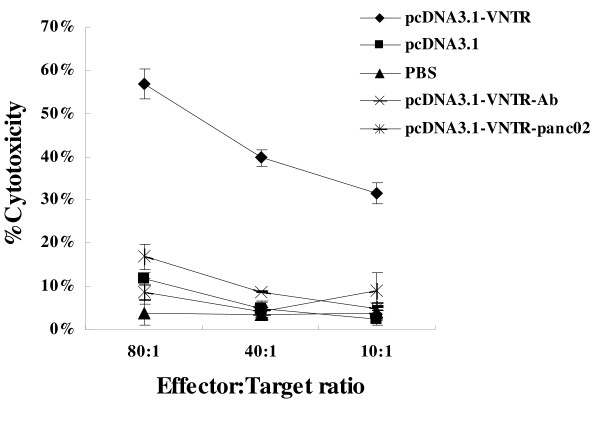
**Induction of cytotoxicity responses**. Splenocytes from immunized mice were isolated 7 days after the third immunization. CTLs were from mice immunized with pcDNA3.1-VNTR, pcDNA3.1 or PBS. Panc02-MUC1or panc02 cells were used as targets. The CTLs induced by the pcDNA3.1-VNTR could specifically kill the panc02-MUC1 cells, but not the panc02 cells and it could be blocked by anti-MUC1 antibody VU3C6.

To determine whether immunization with pcDNA3.1-VNTR could induce strong CTL responses in mice, we carried out the nonradioactive LDH-releasing assay (Figure 6). To measure the specific cytotoxicity, splenocytes from immunized mice were isolated 7 days after the third immunization, and then co-cultured with panc02-MUC1 or panc02 cells at various E/T rations. Splenocytes from mice immunized with pcDNA3.1-VNTR could kill panc02-MUC1, but not panc02, much more efficiently than those from the mice immunized with empty vector or PBS. As shown in Figure 6, when the ratio of E:T was 80:1, effector cells of the pcDNA3.1-VNTR immunized group showed a cytotoxicity of 56.84 ± 3.54% against panc02-MUC1 cells, and when this ratio was 40:1, the cytotoxicity was 39.71 ± 1.97%. This cytotoxicity activity could be inhibited by the anti-MUC1 antibody VU3C6 (Chemicon). However these effector cells had no cytotoxicity activity against the panc02 cells. These results confirmed the superior ability of pcDNA3.1-VNTR to induce MUC1-specific CTL compared to the other groups.

### Therapeutic antitumor immunity

**Figure 7:**
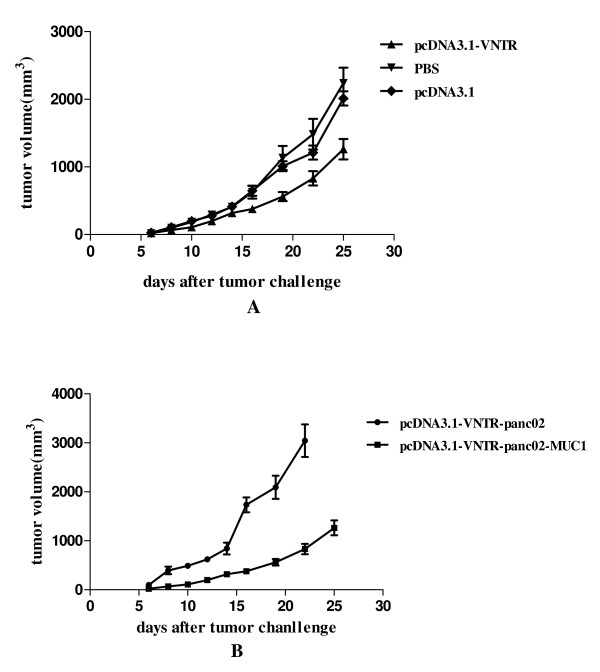
**Induction of therapeutic anti-tumor immunity**. **A: **Panc02-MUC1 tumor growth on day 25 was inhibited significantly in mice immunized with pcDNA3.1-VNTR compared to that in the mice immunized with pcDNA3.1 or PBS (*P *< 0.05). **B:**The panc02 tumor growth was not inhibited in mice treated with the pcDNA3.1-VNTR.

In the therapeutic study, administration of the DNA vaccine started 4 days after the mice injected with a tumorigenic dose of panc02-MUC1 or panc02 cells and were repeated on day 9 and day 13. As shown in Figure 7A, panc02-MUC1 tumor growth on day 25 was inhibited more significantly in the mice immunized with pcDNA3.1-VNTR than in the mice immunized with pcDNA3.1 or PBS (*P *< 0.05). In contrast, the panc02 tumor growth was not inhibited in mice treated with the pcDNA3.1-VNTR, compared with the mice inoculated with panc02-MUC1 cells as shown in the Figure 7B (*P *< 0.05). Furthermore most of the mice inoculated with panc02 died before day 25.

**Figure 8:**
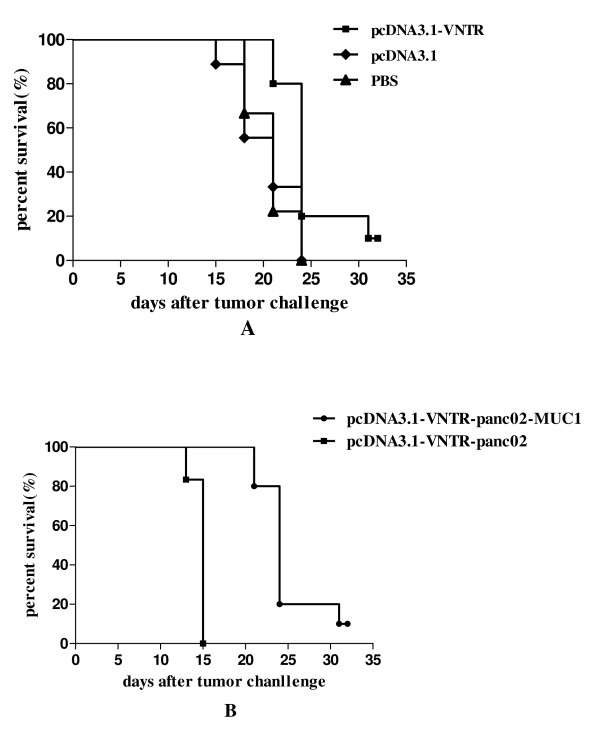
**Effect of DNA vaccine therapy on survival of tumor-bearing mice**. **A: **The panc02-MUC1 tumor bearing mice immunized with panc02-VNTR had a longer survive time than the pcDNA3.1 and PBS groups(*P *< 0.05). **B: **Mice inoculated with panc02 and immunized with pcDNA3.1-VNTR developed lethal tumors and died within 15 days, while the mice inoculated panc02-MUC1 had a longer survive time(*P *< 0.05).

The panc02-MUC1 tumor bearing mice immunized with pcDNA3.1-VNTR survived longer than the mice in the control groups (Figure 8A, *P *< 0.05). However mice inoculated with panc02, and immunized with pcDNA3.1-VNTR had developed lethal tumors and died within 15 days, while the mice inoculated panc02-MUC1 had a longer survive time (Figure 8B, *P *< 0.05). These results demonstrated the inhibitory effect of pcDNA3.1-VNTR vaccine on tumor growth in vivo and indicated that the anti-tumor activity induced by the pcDNA3.1-VNTR was specific for MUC1-expressing tumor cells.

## Discussion

Despite advances in the understanding of pancreatic cancer molecular biology, systemic treatment of this disease remains unsatisfactory. Conventional chemotherapy and radiotherapy have not produced dramatic improvements in response rates or patient survival. Therefore new treatment strategies are clearly needed for this disease.

In 1992, Jonston et al first reported the immunization strategy using the naked DNA vaccine [19]. The injection of naked plasmid DNA into the muscle results in gene expression, which, in return, induces cellular and humoral immune responses against the expressed protein. Compared to the conventional immunization strategy, the DNA vaccine might provide several important advantages. 1) DNA vaccines could induce MHC-|restricted CD8+ T cell response, which may be advantageous compared with conventional protein vaccines.2) DNA vaccines could be manufactured in a relatively less cost manner and stored in relative easy way. 3) DNA vaccines could induce cellular and humoral immunity [20]. Although the DNA vaccination approach seems safe and promising, DNA vaccination with oncogenes still harbors the risk of transformation for the cells that receive and express the oncogenes. MUC1 is a tumor-associated antigen that is over-expressed in pancreatic adenocarcinomas. Although the tumor-associated antigen MUCl could be a potential target molecule for a protective immune response against MUC1-expressing carcinoma cells, tumors are not normally rejected by the host's immune system, despite the fact that MUC1-specific CTLs have been detected in cancer patients. Therefore it is necessary to adjust the MUC1 vaccine to enhance the humoral and cellular immunity.

To investigate the protective and therapeutic effect of the DNA vaccine pcDNA3.1-VNTR against pancreatic cancer, we first constructed a murine pancreatic cancer cell which could express the human MUC1 protein and mainly on the cell membrane (Figure 1 and Figure 2). Although human MUC1 is homologous to murine MUC1, it is still a foreign protein and might induce an immune response from the host itself. In the preventive and therapeutic experiments, however, the differences between pcDNA3.1-VNTR, pcDNA3.1 and PBS groups challenged with panc02-MUC1 are large, as discussed below.

Compared to other MUC1 DNA vaccine, our vaccine pcDNA3.1-VNTR had two specific traits. 1) The optimized Kozak and hMCP-1 sequences were engineered to the start codon region which could enhance the expression of VNTR in muscle cells, thereby inducing more specific CTLs. 2) The vaccine only had one VNTR repeat that could make it much more specific and reduce the passive effect from the other MUC1 sequences [16]. After the injection of pcDNA3.1-VNTR in muscle, there are at least three mechanisms by which the antigen encoded by plasmid DNA is processed and presented to elicit an immune response: 1) direct priming by somatic cells (myocytes, keratinocytes or any MHC-||negative cells); 2) direct transfection of professional antigen presenting cells (APCs); and 3) cross-priming in which plasmid DNA transfects a somatic cells and/or professional APCs and the secreted protein is taken up by other professional APCs and presented to T cells [20]. Our observations indicated that three intramuscular injections of pcDNA3.1-VNTR suppressed, in a MUC1-specific manner, the development of pancreatic cancer expressing MUC1 in C57BL/6 mice (Figure 3). The mice immunized with pcDNA3.1-VNTR followed with the challenge of panc02-MUC1 had a much longer life span than the mice in the control groups (*P *< 0.05, Figure 4). The MUC1 immunotherapy literatures indicate that cellular immune responses are required for tumor protection. Our cytotoxicity assay showed that splenocytes from the pcDNA3.1-VNTR-immunized mice could specifically kill the panc02-MUC1 tumor cells, but not the panc02 tumor cells, and this cytotoxicity could be specifically inhibited by the MUC1 monoclonal antibody VU3C6 (Figure 6). Many studies have shown that the antibody responses to MUC1 play little role in tumor protection, if any [21]. In our experiments, however, anti-MUC1 antibody responses were much higher in the pcDNA3.1-VNTR vaccinated mice, and this may play an important role in mediating tumor protection (Figure 5).

Theoretically, any kind of vaccine should be able to eradicate the tumor. Few studies, however, demonstrated the effect of the MUC1 DNA vaccine on the therapy of pancreatic cancer, and most studies have focused on the preventive effect of the DNA vaccine [22,23]. Gabriele et al [24] conducted a phase I/II clinical trial using human autologous DC transfected with cDNA of the human tumor antigen MUC1 as a vaccine in 10 patients with advanced breast, pancreatic or papillary cancer. Their results showed that immunologic responses could be induced even in patients with advanced disease; yet, long time survival could not be achieved. Ramesh K et al [25] conducted another phase I study of a MUC1 vaccine composed of different doses of MUC1 peptide with SB-AS2 adjuvant in resected and locally advanced pancreatic cancer. Two of 15 resected pancreatic cancer patients achieved disease free for 32 and 61 months at follow-up. Their results showed that MUC1 100 mer peptide with SB-AS2 adjuvant was a safe vaccine that could induce low but detectable mucin-specific humoral and T-cell responses in some patients.

Our study was the first to use the pcDNA3.1-VNTR vaccine, which had a significant therapeutic effect on pancreatic cancer. On day 0 the mice in therapy experiments were inoculated with 1 × 10^6 ^panc02-MUC1 or panc02 cells. Considering that the pancreatic cancer is a very lethal disease and panc02 cells could easily develop tumors in the C57BL/6 mice, the therapeutic immunization was performed 4 days after the inoculation and the mice were immunized 3 times. As shown in the Figure 7A, the panc02-MUC1 tumor volume in the mice immunized with pcDNA3.1-VNTR was much smaller than the mice immunized with pcDNA3.1 or PBS (*P *< 0.05) and the panc02 tumor growth was not inhibited by the immunization with pcDNA3.1-VNTR (Figure 7B, *P *< 0.05). Most of the mice inoculated with panc02 cells died before day25. Survival analysis showed that the panc02-MUC1 tumor bearing mice immunized with pcDNA3.1-VNTR had a longer survival than the control groups (Figure 8A, *P *< 0.05). However mice inoculated with panc02, and immunized with pcDNA3.1-VNTR developed lethal tumors and died within 15 days, while the mice inoculated with panc02-MUC1 survived longer(Figure 8B, *P *< 0.05). These results showed that the pcDNA3.1-VNTR vaccine had a specific therapeutic effect on tumor growth in vivo, and the anti-tumor activity induced by the pcDNA3.1-VNTR was MUC1-specific.

## Conclusion

We constructed a useful MUC1 DNA vaccine. The vaccine was successful in the prevention of tumor establishment and treatment of established tumors in our model. Our data suggested that similar immunization strategies might be used in pancreatic cancer patients with over-expression of MUC1 for the treatment of early cancers or the eradication of minimal residual lesions. Further research is needed to increase the effectiveness of this vaccine.

## Abbreviations

PBS: phosphate buffered saline; VNTR: various tandem repeat units.

## Competing interests

The authors declare that they have no competing interests.

## Authors' contributions

YFR, WCW and XLN co-performed the animal experiments and made the record. DYJ and XYQ co-designed and helped performing the animal experiments and revised the manuscript. WHL, DSW and TTK helped to perform the immunity experiment and helped the statistical analyses. All authors read and approved the final manuscript.

## Pre-publication history

The pre-publication history for this paper can be accessed here:

http://www.biomedcentral.com/1471-2407/9/191/prepub
